# MMP-9 inhibition promotes anti-tumor immunity through disruption of biochemical and physical barriers to T-cell trafficking to tumors

**DOI:** 10.1371/journal.pone.0207255

**Published:** 2018-11-30

**Authors:** Vladi Juric, Chris O'Sullivan, Erin Stefanutti, Maria Kovalenko, Andrew Greenstein, Vivian Barry-Hamilton, Igor Mikaelian, Jeremiah Degenhardt, Peng Yue, Victoria Smith, Amanda Mikels-Vigdal

**Affiliations:** Gilead Sciences, Inc., Foster City, CA, United States of America; Institut national de la recherche scientifique, CANADA

## Abstract

Matrix metalloproteinase-9 (MMP-9), whose expression is frequently dysregulated in cancer, promotes tumor growth, invasion, and metastasis by multiple mechanisms, including extracellular matrix remodeling and growth-factor and cytokine activation. We developed a monoclonal antibody against murine MMP-9, which we found decreased growth of established primary tumors in an orthotopic model of HER2-driven breast cancer (HC11-NeuT) in immunocompetent mice. RNA sequencing (RNAseq) profiling of NeuT tumors and additional mouse model tumors revealed that anti-MMP-9 treatment resulted in upregulation of immune signature pathways associated with cytotoxic T-cell response. As there is a need to boost the low response rates observed with anti-PDL1 antibody treatment in the clinical setting, we assessed the potential of anti-MMP-9 to improve T-cell response to immune checkpoint inhibitor anti-PDL1 in NeuT tumors. Anti-MMP-9 and anti-PDL1 cotreatment reduced T-cell receptor (TCR) clonality and increased TCR diversity, as detected by TCR sequencing of NeuT tumors. Flow cytometry analyses of tumors showed that the combination treatment increased the frequency of CD3+ T cells, including memory/effector CD4 and CD8 T cells, but not regulatory T cells, among tumor-infiltrating leukocytes. Moreover, in vitro enzymatic assays corroborated that MMP-9 cleaves key T-cell chemoattractant CXC receptor 3 ligands (CXC ligand [CXCL] 9, CXCL10, and CXCL11) and renders them inactive in T-cell migration assays. Consistent with our in vitro experiments, analysis of NeuT tumor protein lysates showed that anti-MMP-9 treatment increases expression of CXCL10 and other T cell–stimulating factors, such as interleukin (IL)-12p70 and IL-18. We show that inhibition of MMP-9, a key component of the tumor-promoting and immune-suppressive myeloid inflammatory milieu, increases T-helper cell 1 type cytokines, trafficking of effector/memory T cells into tumors, and intratumoral T-cell diversity.

## Introduction

Tumor growth is influenced by a complex interplay between tumor cells and their microenvironment, including the extracellular matrix (ECM), ECM-associated growth factors, and cytokines, as well as surrounding non-tumor stromal cells and infiltrating immune cells[1]. Numerous reports support a pleiotropic role for matrix metalloproteinase 9 (MMP-9) in promoting tumorigenesis. MMP-9 regulates tumor growth, invasion, and metastasis, including proteolytic remodeling of ECM, alteration of cell-cell and cell-ECM interactions, migration, and angiogenesis[2–9]. Numerous studies have identified the diversity in expression profile, regulation, substrate specificity, and function of MMPs, including those with high primary sequence homology, such as MMP-9 and MMP2[5, 10]. Whereas various MMP family members can sometimes share overlapping expression patterns and substrate specificities, loss of MMP-9, unlike the closely related family members MMP2 or MMP7, is associated with beneficial outcomes in tumor models, suggesting that MMP-9 plays a central role in disease pathology [11].

MMP-9 remodels basement membrane collagen and ECM components, which may facilitate outgrowth and metastasis and promote chemotherapy resistance [12–14]. MMP-9 modulates the function of growth factors and cytokines. It promotes angiogenesis and epithelial proliferation by increasing the bioavailability of ECM-sequestered growth factors such as VEGF, FGF-2, and membrane-tethered EGF [3, 15]. MMP-9 may regulate tumor invasion and metastasis, and promote antitumor immune dysfunction by activating TGF-β [16, 17]. MMP-9-mediated proteolytic cleavage has been shown to activate various tumor-promoting cytokines such as IL-8 and IL-1β [7, 18, 19].

MMP-9 can cleave and potentially inactivate or degrade T-helper cell 1 (Th1)-type chemokines CXC ligand (CXCL) 9, CXCL10, and CXCL11[20–22]. These chemokines act via CXC receptor (CXCR) 3 to stimulate trafficking of CXCR3-positive T cells to tumor sites and associated draining lymph nodes, important steps in antitumor immunity[23]. CXCR3 is rapidly induced on naïve cells following activation, and remains highly expressed, preferentially on Th1-type CD4+ T cells and effector CD8+ T cells. Early studies demonstrated a role for CXCR3 in Th1 and CD8 T-cell trafficking to peripheral sites of Th1-type inflammation and the establishment of Th1-amplification loop mediated by interferon-gamma (IFNγ) and the IFNγ-inducible CXCR3 ligands[24]. CXCR3 plays a role in the migration of T cells to the lymphoid compartment, facilitating the interaction of T cells with antigen-presenting cells and leading to the generation of effector and memory cells[25, 26]. Recent clinical studies associate CXCR3 ligands with response to checkpoint blockade. MMP-9 may hamper antitumor immunity by functionally inactivating CXCR3 ligands.

MMP-9 has been shown to support the development and tumor infiltration of myeloid-derived suppressor cells (MDSCs) through c-kit and osteopontin cleavage, establishing an immunosuppressive microenvironment[27–29]. MMP-9 may antagonize T cell–mediated anti-tumor responses in tumors by shaping a local inflammatory cytokine/chemokine milieu hostile to effector T-cell infiltration, and by increasing the recruitment and/or activation of MDSCs and other suppressive myeloid infiltrates to the tumor.

The combination of the proteolytic breakdown of biochemical barriers to cell invasion, accompanied by growth and angiogenic factor release and the suppression of immune effector cell function, pave the way for tumor expansion.

Immune checkpoint inhibitors such as anti-PD1 (nivolumab or pembrolizumab) and anti-PDL1 (atezolizumab or avelumab) antibodies have demonstrated clinical benefit in several cancers[30, 31]. Inhibition of the PD1/PDL1 axis allows effector T-cell reactivation within the tumor, leading to improved tumor cell killing. Nevertheless, the clinical efficacy of PD1 pathway inhibition as monotherapy has been limited to patient subsets in most tumor types studied to date. Decreasing the levels of immunosuppressive factors that limit T-cell response in the tumor microenvironment, or improving the trafficking of activated, tumor-specific T cells to tumors, may broaden and deepen antitumor responses to PD1/PDL1 inhibition.

We have previously shown that a highly selective and potent allosteric antibody inhibitor of MMP-9 decreased primary tumor growth and metastasis in xenograft mouse models[32]. Melani et al have shown that MMP inhibitor amino-biphosphonate reduces growth of spontaneous tumors in BALB-NeuT mice and reduces MDSC prevalence in tumors[6]. In this report, we explored the mechanism by which MMP-9 promotes tumorigenesis using a variant of this NeuT syngeneic tumor model where MMP-9 is expressed predominantly by myeloid cells [33]. We found that treatment of established tumors with the anti-MMP-9 antibody[32] resulted in upregulation of immune signature pathways in tumors, and that the combination of anti-MMP-9 and anti-PDL1 antibodies increased T-cell receptor (TCR) diversity and frequency of memory/effector T cells in tumors. We corroborated that MMP-9 cleaves and inactivates key T-cell chemoattractant CXCR3 ligands (CXCL9, CXCL10, and CXCL11) in vitro, and that anti-MMP-9 treatment may contribute to tumor T-cell homing/trafficking via stabilization of CXCL10 in vivo. Our data indicate that inhibition of MMP-9, a key component of the tumor-promoting and immune-suppressive myeloid inflammatory milieu, reduces tumor burden and promotes effector/memory T-cell infiltration and diversity when combined with an anti-PDL1 antibody.

## Materials and methods

### Antibody production and purification

Anti-MMP-9 (AB0046) or control immunoglobulin G (IgG) (AB005123) antibodies were prepared as previously described [32]. Anti-PDL1 (AB005690) was cloned from literature reports[34], placed into a mouse IgG1 framework, and expressed in vitro. SEC-HPLC (Tosoh TSK gel G3000SWxl) was used to assess antibody stability and confirm that the antibody preparation contained <5% aggregation (S1 File. Supplemental methods).

### In vivo mouse models

Procedures involving the care and use of animals in the study were reviewed and approved by the Pennsylvania State College of Medicine Institutional Animal Care and Use Committee prior to conduct.

For NeuT animal studies, HC11-NeuT cells expressing an ErbB2 rat homolog were generated by transduction of HC11 mammary epithelial cells with pBabe-puro NeuT retroviral construct. Early-passage HC11-NeuT cells resuspended in serum-free medium, Matrigel, (1:1, v/v) were inoculated into cleared mouse mammary fat pads of 3-week-old syngeneic female Balb/c mice at vivoPharm (Hummelstown, PA). Tumor growth was monitored for 3–4 weeks by palpation, and treatments commenced when mean volume reached 100–200 mm^3^ (day 20). Animals were euthanized 7 days after treatment initiation for pharmacodynamic studies. The efficacy study was terminated on study day 27.

CT26 and LLC PD preclinical studies were carried out by AntiCancer Inc. (San Diego, CA). Female BALB/c or C57/BL mice were obtained from Charles River Laboratories (Wilmington, MA) and were assigned to 4 groups of 15 animals, each based on body weight. Preimplantation tumor stocks of the mouse colorectal cancer cell line CT26 expressing GFP (CT26-GFP) or mouse lung cancer cell line LLC expressing GFP (LLC-GFP) were prepared by subcutaneously injecting tumor cells (concentration, 1–5 × 10^6^ cells/100 μl) into the flanks of nude mice. After expansion, tumor tissues were harvested and cut into fragments of approximately 1 mm^3^. Two such tumor fragments were orthotopically implanted adjacent to the colon (CT26) or to the lung (LLC) of each study animal. For the CT26 study, treatment was initiated 12 days after tumor implantation (mean tumor volume, approximately 100–150 mm^3^). For the LLC study, treatment was initiated 3 days post–tumor implantation. Both studies were terminated 10 days after treatment initiation.

For all in vivo studies, antibodies (mouse control IgG1, anti-MMP-9, or anti-PDL1) were injected intraperitoneally at 20 mg/kg twice per week. A single 50 mg/kg AB0046 loading dose was administered on the morning prior to dosing start. Titer analysis of terminal serum samples confirmed that antibodies achieved acceptable circulating concentrations (S4 Fig).

All animal studies were carried out in strict accordance with the recommendations in the Guide for the Care and Use of Laboratory Animals of the National Institutes of Health, and were approved by the local Institute of Animal Care and Use Committee overseeing each facility where studies were conducted.

### Immunohistochemistry analysis

Sections of formalin-fixed tissue (5 μm) were placed on slides and baked at 60°C for approximately 10 minutes. Staining was performed using the Ventana Discovery Ultra Autostainer and associated reagents (Ventana Medical Systems, 750–601) (S1 File. Supplemental methods), using 100 uL of anti-MMP-9 rabbit monoclonal antibody (Abcam Inc., ab76003) per slide, at a concentration of 0.1 ug/mL. Stained slides were coverslipped using Dako Mounting Medium (Dako, CS703). Images were acquired using the Leica Slide Scanner SCN400 and SCN400 client software (Leica Microsystems Inc., SL801). The digital images were documented by exporting to the Digital Image Hub (DIH-SlidePath).

### Second harmonic image generation

Serial FFPE sections were stained with H&E, Masson’s trichrome, or picrosirius red, or prepared for second harmonic generation (SHG) microscopy. The SHG slides were stained with Sytox orange to mark the nuclei. An antibody to identify epithelial cells (anti–pan cytokeratin, Life Technologies #18–0059) was visualized with an AlexaFluor 633-labeled secondary antibody (Sigma-Aldrich #CF633). The fluorescently stained slides were imaged by multiphoton microscopy to visualize the fibrillar collagen. SHG images were collected on a Zeiss LSM 780 (Carl Zeiss, Jena, Germany) with a Chameleon 2-photon laser (Coherent, Santa Clara, CA). The excitation wavelength was 860 nm, and emission emitted second harmonic light was collected between 410–436 nm. The pan-cytokeratin antibody was observed by excitation at 633 nm and emission between 655–750 nm, while Sytox was observed by excitation at 561 nm and emission between 588–610 nm. All images were uniformly collected with consistent intensity and gain.

### RNA sequencing analysis

RNA samples isolated from frozen tumors using a Qiagen RNeasy kit (Qiagen Sciences Inc., Germantown, MD) were converted into cDNA libraries using the Illumina TruSeq Stranded mRNA sample preparation kit (Illumina #RS-122-2103, San Diego, CA), and RNA sequencing (RNAseq) was performed. The raw fastq files were first run through FastQC to verify the data were of high quality and processed using the Expression analysis mRNAv9-RSEM pipeline. After removing sequencing adapters and other low-quality bases, the clipped fastq files were aligned to the mouse reference genome (build GRCm38) using STAR v2.4. The resulting BAM files were fed into the quantification software, RSEM v1.2.14. RSEM output the counts of the sequencing reads for each gene and sample. After normalization, we performed quality control analyses of the data set to identify strong batch effects and outlier samples using principal component analysis and a sample dendrogram.

Genes with <1 sequencing read count/10^6^ (CPM) in ≥3 samples were removed as low-count genes. Generalized linear regression in edgeR was used to estimate log2 fold changes and p values. The p values were adjusted using the false discovery rate control by following the Benjamini-Hochberg procedure. Next, the estimated p values of all the genes were converted to z scores using the zScores function in the R package gCMAP. The z scores were used to rank the list of genes, which was analyzed with gene set enrichment analysis (GSEA) Preranked included in the Broad GSEA Java tool for gene set enrichment analysis against MSigDB, C2 (curated gene sets), C6 (oncogenic signatures), and C7 (immunologic signatures) collections.

### Q-PCR analysis

Total RNA was isolated from the cells and the iNOS mRNA expression was determined by real-time RT-PCR. The C p value of iNOS was normalized based on that of GAPDH.

Total RNA isolated by EA Quintiles (from fresh frozen NeuT tumor samples) was converted to cDNA using High-Capacity cDNA Reverse Transcription Kit (Thermo Fisher Scientific, Waltham, MA). Quantitative RT-PCR was performed using Viia7 Real-Time PCR System (Applied Biosystems) using TaqMan gene expression assays specific for Cxcl9 (Mm00434946_m1), Cxcl10 (Mm_00445235_m1), Cxcl11 (Mm_00444662_m1), Tgfb1 (Mm01178820_m1), Gzmb (Mm00442834_m1), and Il2ra (Mm01340213_m1). Target gene expression data were normalized to Gapdh (Mm99999915_g1) housekeeping gene.

### Tumor T-cell receptor sequencing

Amplification and sequencing of TCRβ-chain third complementarity-determining regions (CDR3) was performed on tumor-genomic DNA using the immunoSEQ Platform (Adaptive Biotechnologies, Seattle, WA).

Extracted genomic DNA was amplified in a bias-controlled multiplex PCR, followed by high-throughput sequencing on an Illumina instrument[35, 36]. Nucleotide sequences were aligned to the international immunogenetics database (http://www.imgt.org) to annotate V(N)D(N)J gene segments of the receptor. The clonality metric is defined as 1−Pielou’s evenness and is calculated as 1 + Σ*p*_*i*_*log2(p*_*i*_*)*/*log*_*2*_*N* (where *N* is the number of clones and *p*_*i*_ is the clonal frequency of the *i*th clone). Clonality values ranging from 0 (even) to 1 (asymmetric) describe the frequency distribution shape. Data were analyzed using the immunoSEQ Analyzer toolset and plotted using Prism software (GraphPad, v5.01).

### Flow cytometry analysis

Tumors were minced and incubated in digestion buffer (S1 File. Supplemental methods). A single-cell suspension was generated by passing through a 70 μm cell strainer and 2 × 10^6^ cells/sample were used for immunostaining. Nonspecific binding was blocked with rat anti-mouse CD16/CD32 mAb (Fc Block, BD Biosciences, San Jose, CA), containing Live/Dead Aqua (1:100 dilution) (Life Technologies, Carlsbad, CA). Cells were stained with 2 panels of fluorophore-conjugated monoclonal antibodies against T-cell markers (S1 Table). For the T-cell panel, the antibody cocktail was added to cells in the final volume of 100 μL, incubated for 20 minutes on ice, rinsed, and fixed (BD Cytofix, BD Biosciences) for flow cytometry analysis. For the Treg panel, cells were first stained for the same cell surface markers, and fixed/permeabilized for intracellular FoxP3 staining. Data were collected using MACSQuant Analyzer 10 Flow Cytometer (Miltenyi Biotec, Bergisch Gladbach, Germany) and analyzed using FlowJo 10.1r5 software (FlowJo, LLC, Ashland, OR). Each antibody was used at the optimal dilution as determined during prestudy optimization experiments (S2 Table). Singlet, nondebris, viable CD45+ cells were used for analysis. Further gating was performed according to gating strategy (S3 Table).

### Chemokine cleavage assay

Human chemokines CXCL9, CXCL10, and CXCL11 (R&D Systems, Minneapolis, MN) were digested with MMP3-activated MMP-9 in assay buffer (50 mM Tris pH 7.5, 150 mM NaCl, 10 mM CaCL_2_, and 0.05% Brij-35) at 37°C for 16 hours with the indicated enzyme to substrate concentrations. Proteolyzed samples were separated by electrophoresis (12% SDS-PAGE) and analyzed via Western blot (probed with chemokine-specific primary antibodies (R&D Systems) assessed using the Odyssey CLx imaging system (Li-Cor Biosciences, Lincoln, NE). Total protein was visualized using Coomassie blue staining and quantified using the ImageQuant LAS 4000 biomolecular imager (GE Healthcare, Marlborough, MA).

### T-cell chemotaxis assay

Normal human peripheral blood mononuclear cells were separated through Ficoll-Hypaque density-gradient centrifugation from the blood of healthy donors. Blood was obtained from the Stanford blood bank; the blood was not collected specifically for this study and all donors provided written informed consent. T cells were isolated by immunomagnetic negative selection (STEMCELL Technologies, Vancouver, BC, Canada) and activated with IL-2 + CD3/CD28 tetrameric antibody complex (STEMCELL Technologies). For proteolysis of CXCL9, CXCL10, and CXCL11 (R&D Systems), chemokines were incubated with MMP3-activated human MMP-9 with the indicated enzyme to substrate molar ratios for 2 hours at 37°C. Chemotaxis assays were performed in 96-Well Transwell plates (Corning Life Sciences, Corning, NY) with 5 μm pore size, and the bottom wells were loaded with assay buffer alone (0.5% BSA in RPMI) or with assay buffer containing MMP-9-treated or -nontreated CXCL9, CXCL10, or CXCL11. Activated T cells were labeled with Calcein AM (Sigma-Aldrich, St. Louis, MO) for 30 minutes, washed, and resuspended in assay buffer, then loaded on the top of the chemotaxis plate filters at 2 × 10^5^ cells per well. Cells and plates were incubated at 37°C for 6 hours. The top of the chemotaxis plate containing filter and cells was removed and plates were measured with a SpectraMax M5 fluorescent plate reader (Molecular Devices, Sunnyvale, CA) with an excitation wavelength of 485 nm and an emission wavelength of 520 nm.

### Luminex analyses

Tumor lysates were generated by lysing 100 ug of tumor using an OMNI bead ruptor homogenizer (Omni International, Kennesaw, GA) using 1:8 w/v ratio RIPA buffer containing 1X benzonase and protease/phosphatase inhibitors (#CST5872S). After homogenization, samples were centrifuged for 10 minutes at 14K × g at 4°C, the supernatant was aliquoted into new 1.5 mL tubes, and total protein content was measured through BCA analysis. Lysates were analyzed by Ampersand Biosciences (Saranac Lake, NY) via Luminex analysis using the rodent MAP 4.0 mouse panel.

### Graphing and statistical analyses

Data were analyzed and visualized using Prism software. For clinical, histopathological, and immunohistochemistry assessments, the significance of regulation of treatment groups versus the vehicle or control IgG group was assessed using the D’Agostino & Pearson omnibus normality test. Normally distributed data were evaluated by a one-way ANOVA with Dunnett’s Multiple Comparison post-test or with an unpaired t-test with Welch’s correction. Non-normally distributed data were evaluated by either a Mann-Whitney test (for pairwise analysis) or by a Kruskal-Wallis test with the Dunn’s Multiple Comparison post-test. P value designations are as follows: * <0.05, **<0.01, ***<0.001, **** <0.0001.

For fluorescence-activated cell sorting (FACS) analysis, comparison of cells positively stained by antibody at study termination was evaluated by a one-way ANOVA with Dunnett’s Multiple Comparison post-test. Significant differences between individual groups were determined using Sidak’s Multiple Comparison Test. A multiplicity-adjusted p value of ≤0.05 was considered significant.

## Results

### MMP-9 is highly expressed in human tumors

**Fig 1:**
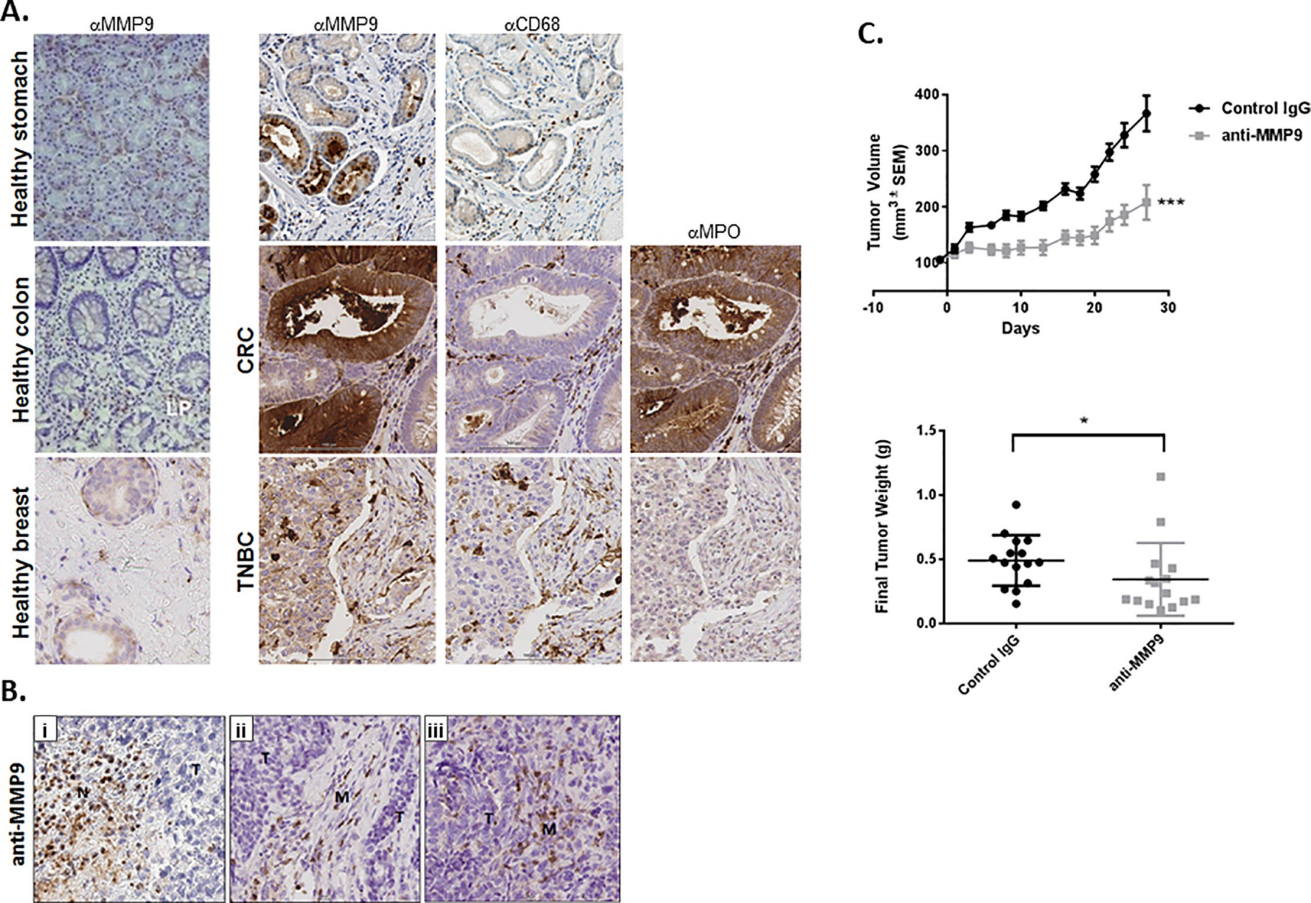
Inhibition of MMP-9 results in decreased tumor growth in a PD1 refractory mouse model. (**A**) MMP-9 expression was assessed in FFPE healthy and tumor (gastric adenocarcinoma, CRC, and triple-negative breast cancer) tissues. MMP-9 was found to be highly expressed in tumor cells and stromal infiltrate as compared to healthy tissue controls. (**B**) IHC analysis of NeuT tumors shows that MMP-9 is predominantly expressed in infiltrating M cells, with limited MMP-9 expression observed in the T cells. (**C**) NeuT tumor cells were injected into the cleared mammary fat pad of recipient mice, and antibody treatment was initiated 33 days postinoculation when tumors reached ~100 mm^3^. Anti–MMP-9 antibody treatment resulted in a significant reduction in tumor volume (p<0.0001 as assessed by one-way ANOVA analysis) and end-of-study tumor weight (p = 0.0164).

MMP-9 is present in a wide variety of tumor types at the mRNA and protein levels. High MMP-9 expression correlates with poor prognosis in many cancers, including gastric[37–39], lung[40, 41], colorectal cancer (CRC)[42, 43], and breast[44, 45]. To confirm these findings and identify the cellular sources and localization of MMP-9 within tumors, we assessed MMP-9 expression in gastric adenocarcinoma, CRC, and triple-negative breast cancer via immunohistochemistry (IHC). We found elevated MMP-9 expression in tumors as compared to normal tissue (Fig 1**A**). MMP-9 expression was heterogeneous, among and within specific tumor types. Positive staining was observed in tumor cells (T). Infiltrating myeloid cells (M), identified by serial costaining with the macrophage marker CD68 and the neutrophil marker myeloperoxidase, showed high MMP-9 expression (Fig 1A).

#### Anti–MMP-9 antibody treatment reduces tumor growth in a syngeneic, orthotopic mouse tumor model

We have previously shown that specific MMP-9 inhibition with a monoclonal antibody reduces primary tumor growth and metastasis in a xenograft model of CRC.[32] The anti–MMP-9 antibody has been previously shown to specifically block pro–MMP-9 activation and MMP-9 enzymatic activity while potentially leaving the nonenzymatic signaling activity of MMP-9 intact.[46] However, antitumor immune response could not be investigated in the CRC model due to the impaired adaptive immune system of the nude mice.

To study the effects of MMP-9 inhibition on tumorigenesis in the context of a fully functional immune system, we used the HC11-NeuT syngeneic orthotopic mouse model of breast cancer.[33] IHC assessment of MMP-9 expression in established NeuT tumors revealed that, in contrast to the broad MMP-9 staining observed in human tumors, mouse MMP-9 expression was restricted to neutrophils in areas of necrosis (Fige 1Bi) and to macrophages in the tumor capsule region (Fig 1Bii). Tumor cells appeared to be largely MMP-9-negative. (Fig 1Biii). To assess the role of MMP-9 in the NeuT model, animals with established tumors were treated with control IgG or anti-MMP-9 antibody. Notwithstanding the relatively limited MMP-9 expression observed in these tumors as compared to human disease, anti–MMP-9 antibody treatment significantly reduced tumor growth as compared to IgG control (56% vs 335% tumor volume increase, respectively; p = 0.0005). Tumor weight in the anti–MMP-9–treated group was reduced compared to the control (Fig 1C). Thus, MMP-9 inhibition decreases tumor growth in both xenograft and syngeneic model settings.

#### Anti–MMP-9 antibody treatment increases immune pathway gene expression in tumors

**Fig 2:**
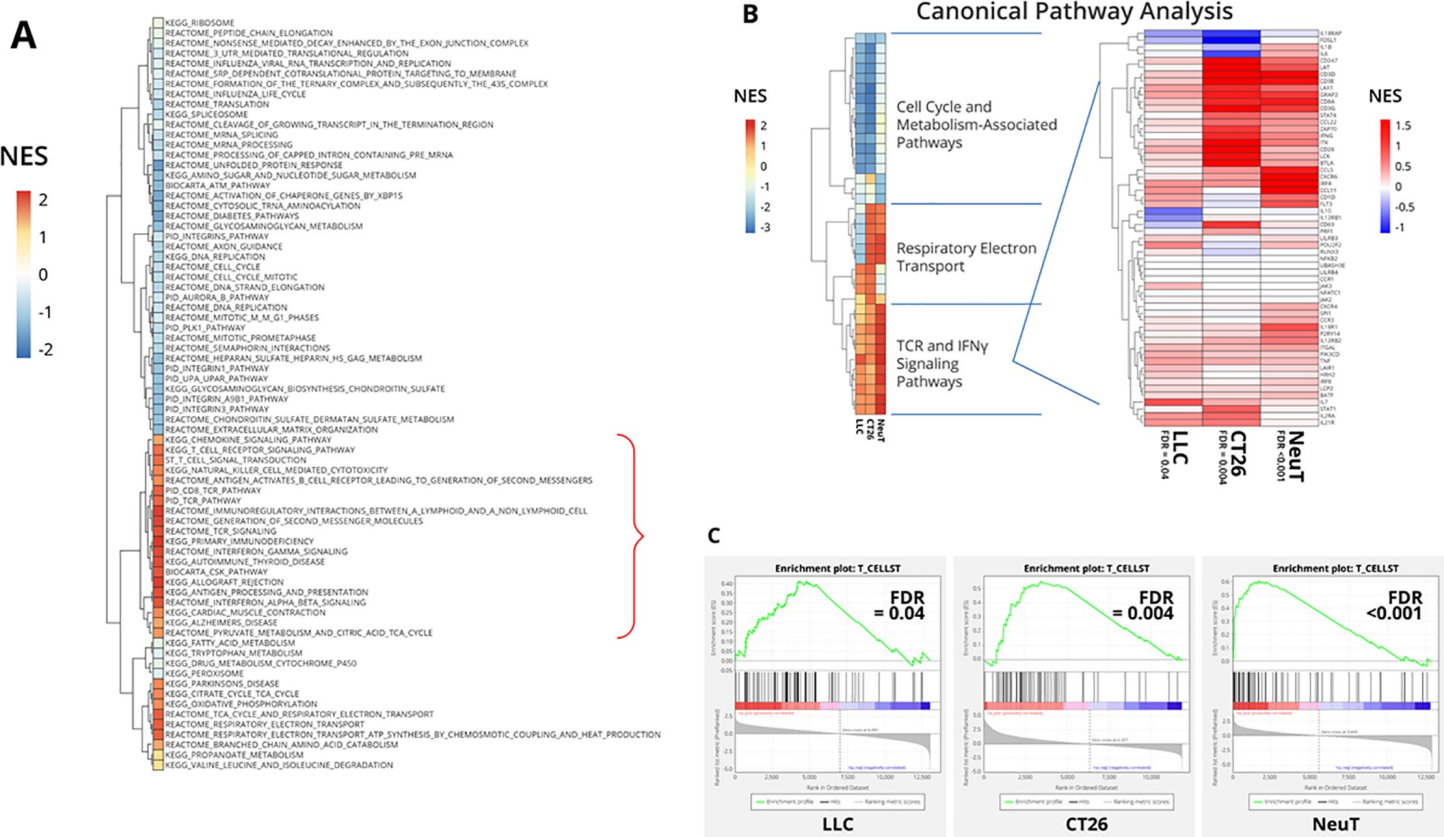
MMP-9 inhibition elevates expression of genes associated with effector T-cell activation pathways in independent models. (**A**) RNAseq gene expression analysis of end-of-study NeuT tumors (n = 15/group; day 27) indicates that anti–MMP-9 treatment results in upregulation of T-cell activation signatures within the tumors as compared to control IgG treatment. (**B**) A comparison of RNAseq analysis of 3 independent tumor models (LLC, CT26, and NeuT) showed consistent alterations in gene expression in T-cell signature pathways mediated by anti–MMP-9 antibody treatment as compared to control IgG. (**C**) The T-cell signature pathway was consistently induced by anti–MMP-9 antibody treatment, indicative of an increased Th1 immune response.

To address the mechanism of action of MMP-9 in NeuT tumors, we performed RNAseq gene expression analysis on tumor samples taken at study end (day 27). GSEA revealed upregulation of immune signature pathways and downregulation of ECM signatures in tumors treated with anti–MMP-9 (Fig 2A and Panel A in S1 Fig).

Since anti–MMP-9 reduced ECM signature in tumors, we performed SHG microscopy, a multiphoton method that specifically visualizes crystalline fibrillar collagen[47], to assess the effects of anti–MMP-9 treatment on fibrillar collagen composition within the tumors. Cytokeratin staining for tumor cells revealed that the tumors grew heterogeneously, both in large masses and in small acinar structures. Collagen fibrils in and around both structures appeared to be diminished in the anti–MMP-9–treated tumors as compared to the isotype control treated group (Panel B in S1 Fig). Fibrillar-collagen intensity quantification across entire images confirmed a trend toward decreased fibrillar collagen present in the anti–MMP-9–treated tumors, although this did not reach statistical significance. This trend in reduced collagen was consistent with SHG imaging from an additional mouse model (HCT116 xenograft model) wherein anti–MMP-9 antibody treatment has previously been shown to be effective[32] and suggests that inhibition of MMP-9 enzymatic activity may affect fibrillar collagen networks around different types of tumors (Panel C in S1 Fig).

RNAseq data analysis of immune pathways identified key immune signatures upregulated by anti–MMP-9, including T-cell and IFN (IFNγ and IFNα/β) signaling signatures (Fig 2A). Meta-analysis of RNAseq data from the NeuT tumors and 2 additional syngeneic mouse models, CT26 and LLC (see S2 Fig for in-life data), revealed that anti–MMP-9 consistently promoted activation of T-cell receptor (TCR) and IFNγ signatures as compared to IgG control antibody treatment (Fig 2B). Examination of the T-cell signature enrichment plots across the various models showed the majority of the genes within this signature were upregulated in response to anti–MMP-9 treatment (FDR <0.05, FDR <0.001, and FDR <0.005 for LLC, NeuT, and CT26 models, respectively) (Fig 2C). Taken together, these data indicate that inhibition of MMP-9 in immune-competent mice generally upregulates immune signatures associated with a Th1-type immune response.

#### Anti–MMP-9 in combination with anti-PDL1 antibody treatment reduces tumor T-cell clonality, increases T-cell diversity

**Fig 3:**
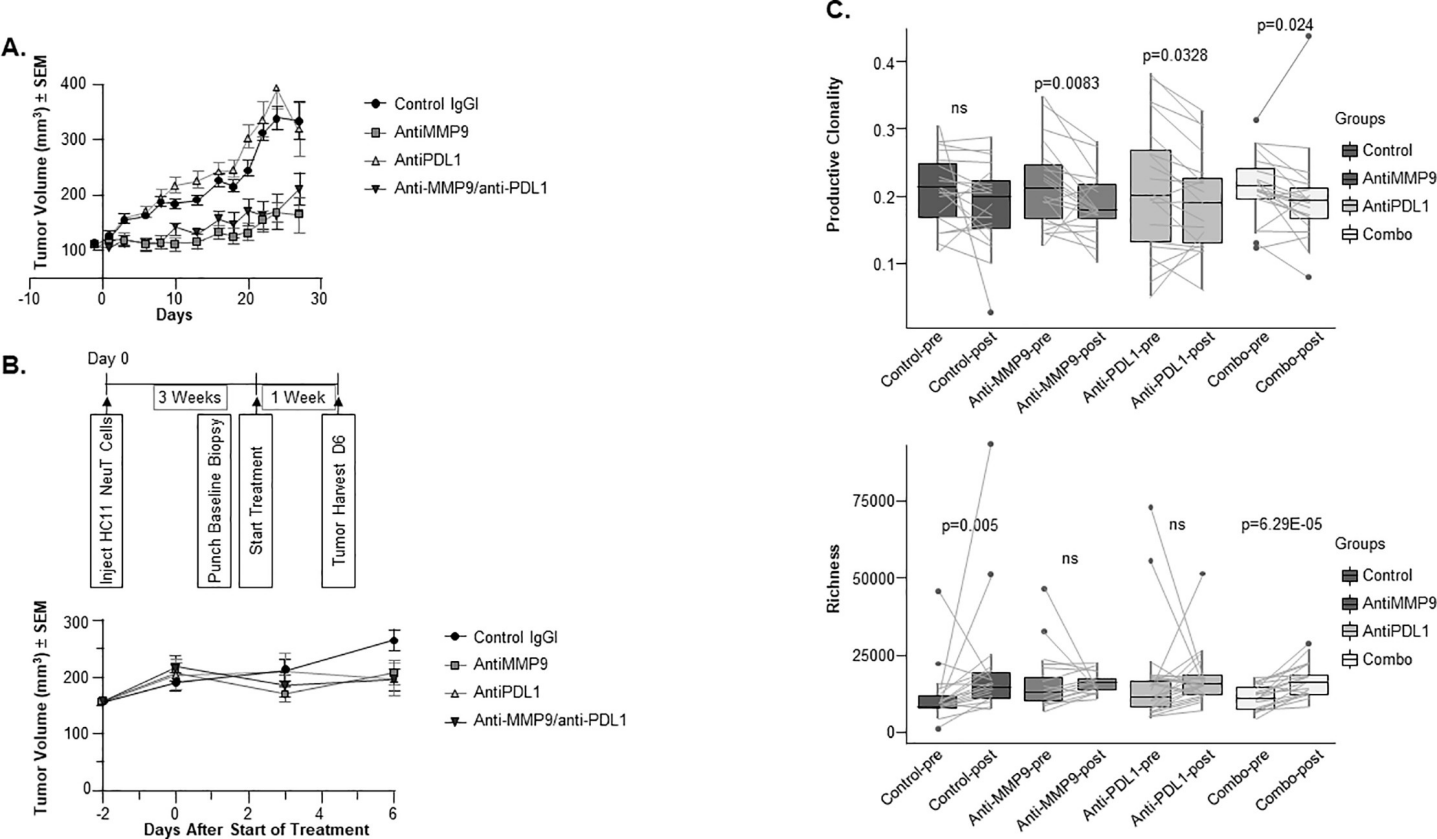
Anti–MMP-9 anti-PDL1 antibody combination results in tumor growth inhibition associated with increased TCR diversity. (A) NeuT tumor cells were injected into the cleared mammary fat pad of recipient mice and antibody treatment was initiated 33 days postinoculation when tumors reached ~100 mm^3^. Animals were treated with control IgG, anti–MMP-9, anti-PDL1, or the combination for 27 days (n = 15/group). (B) NeuT tumors were implanted into mammary fat pads of recipient mice and grown until tumors reached ~100 mm^3^ (day 20), at which point pretreatment biopsies were collected. Animals were treated for 6 days with anti–MMP-9, anti-PDL1, or the combination, then tumors were collected and analyzed by TCR sequencing (n = 20/group). No significant differences in tumor volumes were associated with the various treatment groups on study day 6. (C) TCR sequencing revealed that anti–MMP-9 (p = 0.0083), anti-PDL1 (p = 0.0328), and the combination (p = 0.024) resulted in decreased TCR clonality between tumors at day 0 and day 6 (upper panel, n = 20/group). Anti–MMP-9 and anti-PDL1 combination treatment also led to increased TCR richness (p = 6.29E-05), suggestive of a more diverse T-cell repertoire (lower panel, n = 20/group). Representative clonality and diversity readout from 2 independent TCRb sequencing studies using tumor gDNA samples from 2 independent cohorts of mice are shown.

Since anti–MMP-9 increased immune signatures and decreased ECM signatures in tumors, we hypothesized that it may act by promoting T-cell trafficking and/or infiltration to tumors. This would make anti–MMP-9 a suitable combination partner with PD1/PDL1 checkpoint inhibitors[48]. In the NeuT tumor model, however, anti-PDL1 treatment alone neither exerted antitumor efficacy nor further potentiated the efficacy of anti–MMP-9 in combination (Fig 3A). The same anti-PDL1 antibody reduced tumor growth in an alternative mouse model (RENCA; Panel A in S3 Fig), suggesting that the NeuT model is largely refractory to PDL1 inhibition. Considering the potent myelosuppression reported in this model[28, 49], the lack of anti-PDL1 efficacy may be due to inhibition of effector T-cell function by myeloid suppressor cells[50]. Although anti-PDL1 did not demonstrate antitumor efficacy in this model, it promoted gene expression changes in tumors detected by RNAseq, such as increases in cell cycle pathway components and immune signatures, and decreased expression in TCA cycle pathway factors (Panel B in S3 Fig).

To evaluate the mechanism of action of anti–MMP-9 with regard to T-cell signaling signatures, we assessed the repertoire of tumor-infiltrating T cells by TCRβ sequencing. We compared TCR sequences from the same tumors before treatment (baseline punch biopsies) and after a short-term treatment with antibodies (day 6 biopsies). The 6-day time point was selected to optimally assess T-cell infiltration while minimizing antitumor efficacy as a confounding factor in the analysis. No significant differences in tumor volumes were associated with the various treatment groups on study day 6 (Fig 3B). Anti–MMP-9 and anti-PDL1 treatment, either alone or in combination, decreased T-cell clonality in tumors (Fig 3C). Furthermore, tumors treated with the combination of anti–MMP-9 and anti-PDL1 antibodies had significantly higher richness, as estimated by the Daley-Smith richness metric implemented by Adaptive Technologies[51] (Fig 3C). Thus, we concluded that the anti–MMP-9 antibody cooperates with anti-PDL1 antibody to achieve a more diverse T-cell repertoire within the NeuT tumors.

#### Combination treatment of anti–MMP-9 and anti-PDL1 antibodies promotes an increase in effector memory CD4+, and CD8+ T cells in tumors

**Fig 4:**
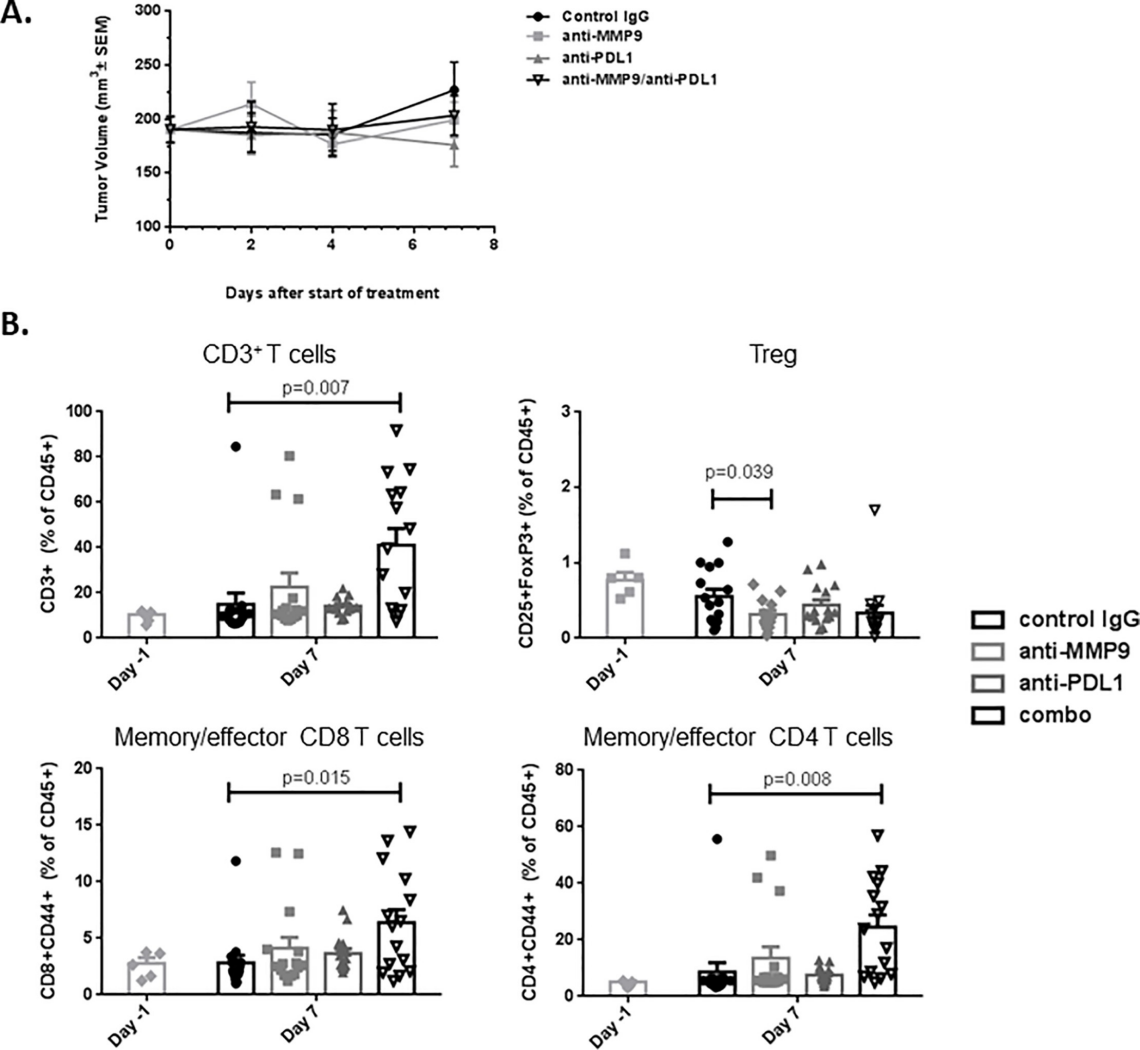
Anti–MMP-9, anti-PDL1 combination promotes increase in fraction of memory/effector CD4 and CD8 T cells. NeuT tumors isolated pretreatment and 7 days post-treatment with control IgG, anti–MMP-9, anti-PDL1, or the combination were analyzed by flow cytometry for immune cell markers. (**A**) The various treatments showed no significant effect on tumor growth at the time point examined (n = 15/group). (**B**) Combination of anti–MMP-9 and anti-PDL1 antibody treatment increased the proportion of CD3+ T-cells, including memory/effector CD8 and CD4 T-cells (CD3+CD8+CD44+ and CD3+CD4+CD44+, respectively) among tumor-associated leukocytes (CD45+ cells). Tregs (CD3+CD4+CD25+FoxP3) were not increased by combination treatment (n = 15/group). The experiment was done 3 times using 3 different mouse cohorts, yielding similar results; representative T-cell phenotyping data is shown.

While the TCR sequencing analysis suggested that combination treatment promotes infiltration and expansion of new T-cell clones, it cannot discriminate between different T-cell subtypes. We performed T-cell phenotyping by flow cytometry on tumors treated in the short term (7 days) with control IgG, anti–MMP-9, anti-PDL1, or the combination. No significant differences in tumor volume were observed among treatment groups at this time point, ensuring lack of bias due to tumor size (Fig 4**A**).

Cotreatment with anti–MMP-9 and anti-PDL1 antibodies increased the proportion of CD3+ cells among tumor-infiltrating leukocytes (Fig 4B), consistent with the observed increase in TCR diversity (Fig 3C). The same treatment group had a larger proportion of CD4+ T and CD8+ memory/effector T cells (CD3+CD4+CD44+ and CD3+CD8+CD44+, respectively) among tumor-infiltrating leukocytes, suggesting that the anti–MMP-9/anti-PDL1 combination promotes a functional T cell–mediated antitumor response. Importantly, while the treatment increased memory/effector T cells, it did not increase Tregs (CD3+CD4+CD25+FoxP3+) (Fig 4B), the frequency of which was low overall in these tumors. Taken together, 3 independent analyses—including gene-expression profiling, TCR sequencing, and flow cytometry—suggest that inhibition of MMP-9 reduces NeuT tumor growth in part by increasing T-cell diversity and memory/effector function.

#### MMP-9 inactivates key T-cell trafficking chemokines CXCL9, CXCL10, and CXCL11

**Fig 5:**
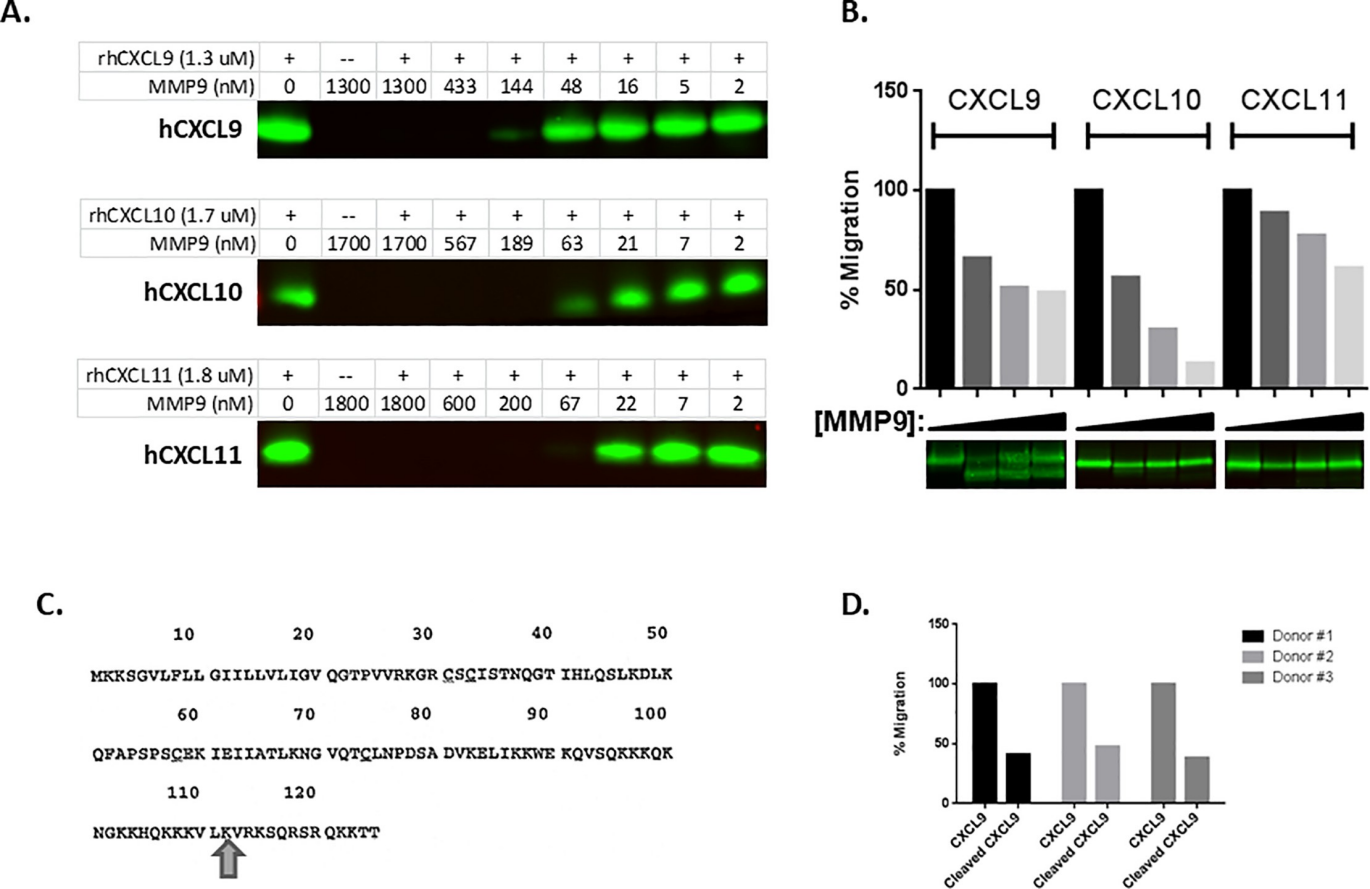
MMP-9 cleaves effector T-cell chemoattractant ligands in vitro, renders them functionally inactive in T-cell migration assays. (**A**) Overnight treatment of CXCL9, CXCL10, and CXCL11 proteins with activated MMP-9 results in protein degradation in a dose-responsive manner (**B**) Transient (2 hours) cleavage of CXCL9, CXCL10, and CXCL11 with activated MMP-9 renders the chemokines functionally inactive in primary, activated T-cell migration assays across multiple donors. A representative result of 3 independent experiments is shown. (**C**) MMP-9 cleaves CXCL9 at the C-terminus at a previously described cut site. (**D**) Assessment of the activity of truncated CXCL9 protein shown in (**C**) revealed significantly reduced activity compared to wild-type CXCL9 in the activated T-cell migration assay across multiple donors.

We next sought to determine whether MMP-9 regulates T-cell trafficking into tumors. CXCR3 ligands CXCL9, CXCL10, and CXCL11, which promote selective trafficking of activated Th1-type CD4 T cells and effector CD8 T cells to tumors[25, 26], are reported to be substrates of various proteases[21, 22, 52]. As we could not reliably detect these chemokine breakdown products in the murine tumor model, we performed an analysis of human chemokine processing by human MMP-9. We performed in vitro cleavage assays using MMP3-activated MMP-9 protein incubated overnight with the CXCR3 ligands and confirmed that MMP-9 digests all 3 proteins in a dose-dependent manner (Fig 5A).

The MMP-9-cleaved chemokines were further analyzed in primary human T-cell chemotaxis assays to assess the functional consequences of MMP-9 cleavage. We found that a transient incubation (2 hours) of CXCL9, CXCL10, and CXCL11 with activated MMP-9 prevented their ability to promote T-cell chemotaxis in a dose-dependent manner (Fig 5B). This functional inhibition of CXCL9, CXCL10, and CXCL11 by MMP-9 was seen in multiple blood donors, in at least 3 different independent assays, performed on different days. Using a cutoff of 30% inhibition of T-cell migration, 8 of 10, 8 of 9, and 4 of 10 donors showed similar responses for MMP-9-cleaved CXCL9, CXCL10, and CXCL11, respectively.

Transient treatment of CXCL9 with active MMP-9 resulted in the emergence of a lower molecular weight species (at the expense of the full-length protein), which was further examined via mass spectrometry. Incubation with activated MMP-9 results in the formation of a C-terminal cleavage product, previously shown to be an inactive species of CXCL9 (Fig 5C)[52]. We expressed this cleaved product and found it had significantly less activity in promoting migration of activated primary T cells (Fig 5D) than wild-type CXCL9 protein in the T-cell migration assays.

#### MMP-9 inhibition promotes expression of chemokines associated with antitumor immunity and T-cell trafficking

**Fig 6:**
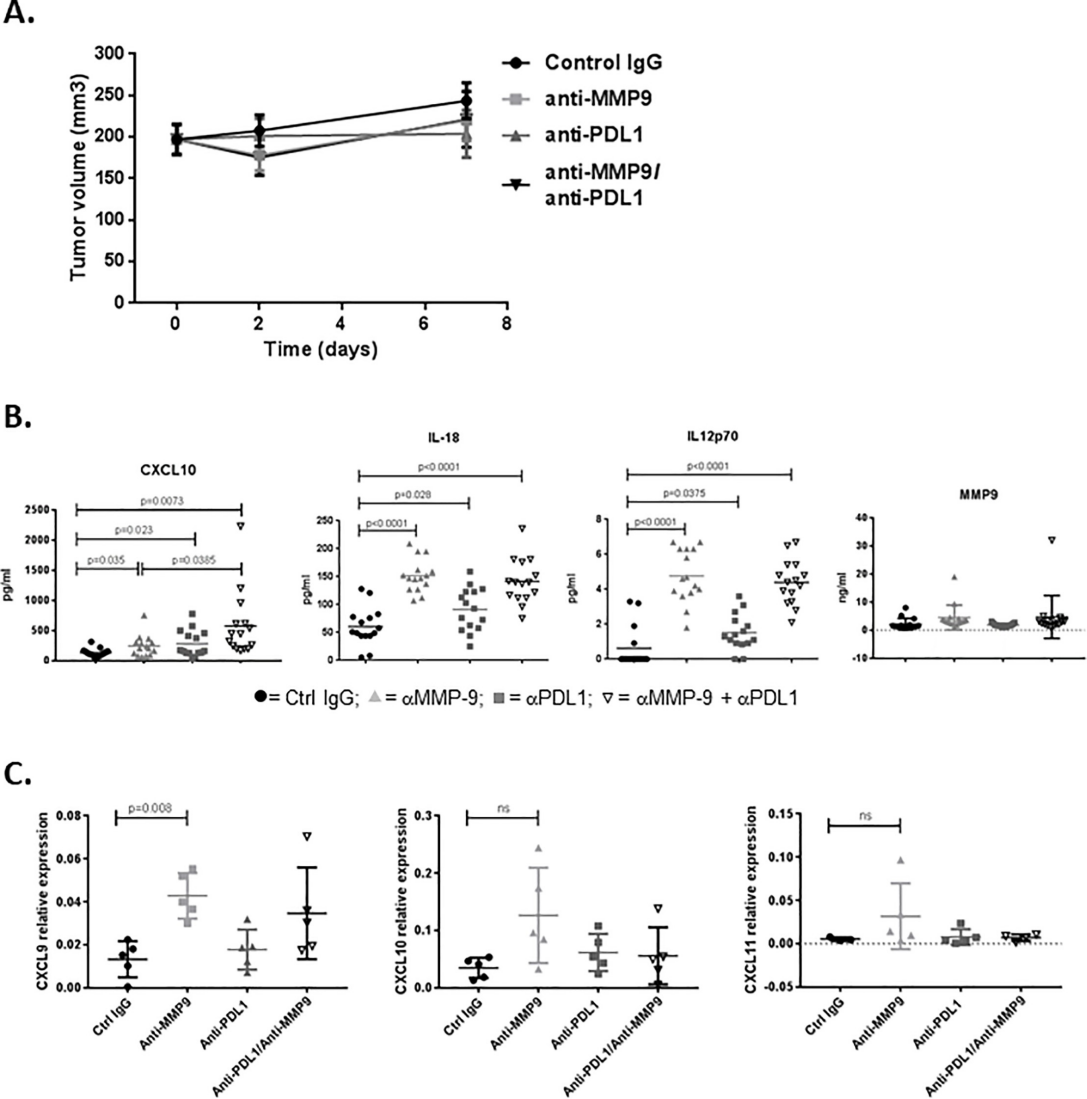
Anti–MMP-9 treatment increases levels of proteins associated with activated T-cell trafficking and immune activation. Mice bearing NeuT tumors (~100 mm^3^) were treated for 7 days with control IgG, anti–MMP-9, anti-PDL1, or the combination, at which point tumors were collected, lysed, and analyzed for protein expression via Luminex. (**A**) The various treatments showed no significant effect on tumor growth at the time point examined (n = 15/group). (**B**) Luminex analysis revealed increased levels of T-cell trafficking (CXCL10) and immune cell activation (IL-12 and IL-18) factors by anti–MMP-9, while MMP-9 protein levels remained unchanged by any of the treatments (n = 15/group). (**C**) mRNA samples (n = 5/group) were analyzed by qPCR for CXCL9, CXCL10, and CXCL11, and confirmed the expression results shown in Fig 2. The same mRNA was used for qPCR and RNAseq.

Finally, to assess protein expression changes in NeuT tumors after short-term treatment (7d) with anti–MMP-9, anti-PDL1, or the combination treatments, we performed Luminex analysis on tumor lysates. Again, no significant differences in tumor volume were observed after 7 days of treatment (Fig 6A). Consistent with our in vitro results, CXCL10 protein levels were significantly increased upon anti–MMP-9 antibody treatment in vivo and were further increased in the combination setting (Fig 6B; p<0.05 and p<0.01, respectively). In addition, expression of proteins associated with anti-tumor immunity (eg, IL-12 and IL-18) was increased upon anti–MMP-9 and anti-PDL1 antibody combination treatment. No statistical differences were observed between anti–MMP-9 antibody treatment and the combination for these 2 markers. We also found that MMP-9 protein levels in tumors were unchanged by anti–MMP-9 treatment (Fig 6B), suggesting that the antibody exerts its effects by enzymatic inhibition of this protease, without reducing the level of MMP-9 in tumors.

Overall, these data support the hypothesis that combined inhibition of MMP-9 and PDL1 drives a Th1-type anti-tumor immune response by decreasing physical ECM barriers and increasing protein levels of Th1-type cytokines, trafficking of effector-memory T cells into tumors, and boosting intratumoral T-cell diversity.

## Discussion

Increased MMP-9 expression is observed in a wide range of tumor types, including gastric, CRC, and breast cancer in diverse cell types, and is associated with poor outcomes[37, 39–41, 43–45, 53]. In this report, we have shown, through multiple independent lines of evidence, that MMP-9 inhibition in preclinical mouse models can promote anti-tumor immunity by altering physical and biochemical properties of tumors relevant to effector T-cell trafficking.

We previously evaluated the effects of inhibiting MMP-9 on primary tumor growth and metastasis in a CRC mouse xenograft model, HCT116[32]. Those studies revealed that targeting both tumor- and stroma-derived MMP-9 resulted in anti-tumor benefits. In the current study, we focused on the use of the syngeneic NeuT tumor model to assess the effects of MMP-9 inhibition on the adaptive immune response.

The ECM is commonly dysregulated and disorganized in cancer but can act as a physical barrier to T-cell infiltration, preventing T-cell interaction with tumor cells[54]. In the NeuT mouse model, we observed that MMP-9 inhibition reduced expression of ECM components. It is plausible that the fibrillar collagen reduction is due to TGF-β signaling decrease by anti–MMP-9, as MMP-9 is thought to promote fibrosis through proteolytic activation of latent TGF-β[4]. The concomitant increase in T-cell activation signature in tumors suggests that anti–MMP-9 may act to inhibit the production of fibrillary collagen and thus reduce physical barriers to T-cell infiltration.

Strikingly, anti–MMP-9 also promoted an increase in immune activation pathways (T-cell activation, IFNγ, Th1) identified by RNAseq in multiple mouse syngeneic models. We evaluated several mechanisms that could lead to increased immune activation signatures in tumors. The above-mentioned effect of anti–MMP-9 on reduction of fibrillar collagen in tumors could be one mechanism, which allows improved access of T cells into tumors.

We corroborated by biochemical and T-cell migration assays that MMP-9 cleaves and functionally inactivates CXCL9, CXCL10, and CXCL11 chemokines, which are chemotactic for CXCR3-expressing activated Th1 T cells and effector CD8 T cells[25, 26]. Thus, we postulate that MMP-9 limits anti-tumor response, at least in part, through degradation of tumoral CXCR3 ligands, resulting in suboptimal trafficking of Th1 cells to tumors. Once MMP-9 is inhibited, active CXCR3 ligands would accumulate in tumors and tumor-draining lymph nodes and promote trafficking of new Th1 CD4 T cells and effector CD8 T cells into tumors. This activity of the anti–MMP-9 antibody could also be contributing to the increased immune signatures in tumors, and is of notable translational relevance given that CXCR3 ligands in the tumor microenvironment are associated with response to adoptive transfer and checkpoint therapies[23–26, 38]. Indeed, protein expression analysis of NeuT tumors revealed that CXCL10 protein levels were elevated in tumors from anti–MMP-9–treated mice. Since CXCL10 transcript levels did not change by anti–MMP-9 treatment, we suspect that anti–MMP-9 treatment stabilizes CXCL10 chemokine.

Even though our studies of the mechanism of anti–MMP-9 action in tumors have converged on chemokine processing, we cannot exclude a possibility that anti–MMP-9 improves effector T-cell function and recruitment to tumors by other mechanisms. It is presently unknown whether this allosteric anti–MMP-9 antibody compromises the function of hemopexin domain of MMP-9 important for MMP-9 localization and substrate specificity [55, 56], and whether this domain is relevant for regulation of anti-tumor T-cell mediated response in the first place. While there is ample evidence on the role of MMP-9 in promoting thymic lymphoma growth [57, 58], less is known about the effects of MMP-9 on normal T-cell function. Our study contributes to this knowledge by connecting, indirectly, the catalytic function of MMP-9 to regulation of T-cell response in tumors. We showed that MMP-9 inhibition coupled with immune checkpoint blockade (eg, anti-PDL1) may be an effective anti-tumor treatment, as the combination resulted in increased TCR diversity and Th1 response in tumors. The increase in TCR diversity was accompanied by an increase in tumoral memory/effector T cells, including both CD4 and CD8 T cells, while there was no increase in Tregs, as determined by flow cytometry. These data suggest that anti-MMP-9/anti-PDL1 combination treatment rebalanced the T-cell milieu within the tumors in favor of more diverse, effector anti-tumor immunity.

While the NeuT model has many benefits (syngeneic tumors grow in immunocompetent animals, tumor growth is driven by a relevant oncogene, the model displays a myeloid infiltrate consistent with human disease, etc), it has several limitations. The strong anti–MMP-9 efficacy observed precluded the assessment of the combined anti–MMP-9/anti-PDL1 impact on tumor growth. We focused on performing shorter pharmacodynamic studies to better understand the joint mechanism of these 2 therapies.

We saw a memory/effector T-cell signature in the tumors of mice treated by anti–MMP-9 and anti-PDL1. Rechallenge experiments attempting to assess the effects of MMP-9 inhibition on immunological memory were not feasible as no NeuT tumor regrowth was observed in those mice, perhaps due to the age of the animals or the potential for immunological memory to the rat NeuT antigen protein. Further, we were unable to definitively determine the long-term consequences of anti–MMP-9 antibody treatment on anti-tumor immunity using this model. While we sampled entire tumors for FACS and TCR sequencing analyses, we observed high mouse-to-mouse variability. When assessing the TCR diversity pre- and postantibody treatment, high variability in the control IgG treatment group may have contributed to the statistically significant increase observed. In contrast, the anti–MMP-9/anti-PDL1 combination treatment group showed reduced variability, strengthening the findings that combination treatment increased TCR diversity in a biologically meaningful way.

The anti–MMP-9 monoclonal antibody andecaliximab is under clinical evaluation and has already produced promising results in early-phase studies of patients with gastric or gastroesophageal tumors[59–61]. The anti-PD1 antibodies pembrolizumab and nivolumab have recently been approved by regulatory agencies for use in various gastric cancer settings. These anti-PD1 agents increased patient survival, but overall response rates have remained low (~11%–15%)[59, 61], pointing to a need to boost overall response and progression-free survival in this setting. Based on the preclinical work we presented, the combination of anti–MMP-9 with inhibitors of the PD1 axis may be a novel approach that potentially addresses that need. Its efficacy is currently being assessed in phase 2 clinical trials (NCT02864381).

## Supporting information

S1 Table Fluorophore-conjugated monoclonal antibodies against T-cell markers used in the flow cytometry analyses. (DOCX)

S2 Table Specifics of use for the antibodies used in the flow cytometry analyses. (DOCX)

S3 Table Flow-cytometric gating strategy for each T-cell marker. (DOCX)

S1 Fig GSEA reveals pathways altered in response to anti-MMP9, anti-PDL1, or anti-MMP9 plus anti-PDL1 combination antibody treatment. (**A**) Analysis of the top ten pathways modulated by anti-MMP9 revealed upregulation of pathways associated with immune activation and downregulation of ECM pathways (*black arrows*). (**B**) Consistent with RNAseq data, second harmonic imaging analysis of end-of-study tumors revealed that anti-MMP9–treated tumors showed a trend for decreased fibrillar collagen content as compared to control IgG–treated tumors. Figure shows results of analysis of the top 10 pathways altered by anti-PDL1 antibody treatment alone.
(TIF)

S2 Fig CT26 and LLC models. (**A**) Tumors from the CT26 orthotopic model were collected on day 10 for RNAseq analysis. Anti-MMP9 treatment showed no significant effects on tumor size at this time point. Tumor volume over the course of study (*left panel*); tumor weight at termination (*right panel*). (**B**) LLC tumors were collected at the end of study (day 14) for RNAseq. Mice treated with anti-MMP9 antibody showed a significant decrease in final tumor weight as compared to control IgG or vehicle treatment at time of gene expression analysis (p = 0.05).
(TIF)

S3 Fig Anti-PDL1 antibody treatment is effective in the RENCA syngeneic tumor model and has effects on tumor gene expression in the NeuT model. (**A**) Female BALB/c mice were injected orthotopically with RENCA tumor cells. Five days post-injection, animals were treated with 20 mg/kg anti-PDL1 twice weekly for 19 days. Treatment with anti-PDL1 antibody resulted in significant tumor growth reduction at the end of the study (p = 0.002). (**B**) RNAseq GSEA analysis of end-of-study NeuT tumor samples from study shown in Fig 3A.
(TIF)

S4 Fig Pharmacokinetics of antibodies used in the 27-day efficacy study. Serum was collected at the end of the study presented in Fig 3A. Both antibodies were found to be at sufficient levels to inhibit their respective antigen targets.
(TIF)

S1 File Supplemental methods. (DOCX)

S2 File Supplemental table. (XLSX)
