# Supplementary material for: MMP-9 inhibition promotes anti-tumor immunity through disruption of biochemical and physical barriers to T-cell trafficking to tumors
Source: PLoS One. 2018 Nov 30;13(11):e0207255. doi: 10.1371/journal.pone.0207255 (PMC6267998; doi:10.1371/journal.pone.0207255)
Supplement: S1 Table — (DOCX) [file pone.0207255.s001.docx]

**Supplemental Table S1. Fluorophore-conjugated monoclonal antibodies against T-cell markers used in the flow cytometry analyses**

| **Antigen** | **T-cell Panel** | **Treg Panel** |
| --- | --- | --- |
| CD45 | PE-Cy7 | PE-Cy7 |
| CD3ε | APCe780 | APCe780 |
| CD4 | PE | PE |
| CD8a | PE-Cy5 |  |
| CD44 | eFluor450 | eFluor450 |
| CD25 | Alexa700 | Alexa700 |
| FoxP3 |  | PE-Cy5 |

APC = Allophycocyanin; PE = R-Phycoerythrin
