# Supplementary material for: MMP-9 inhibition promotes anti-tumor immunity through disruption of biochemical and physical barriers to T-cell trafficking to tumors
Source: PLoS One. 2018 Nov 30;13(11):e0207255. doi: 10.1371/journal.pone.0207255 (PMC6267998; doi:10.1371/journal.pone.0207255)
Supplement: S2 Table — (DOCX) [file pone.0207255.s002.docx]

**Supplemental Table S2. Specifics of use for the antibodies used in the flow cytometry analyses**

| **Antibody** | **Clone** | **Isotype** | **Fluorochrome** | **Quantity Used**  **(µL/sample)** | **Supplier** |
| --- | --- | --- | --- | --- | --- |
| CD45 | 30F11 | Rat IgG2b, κ | PE-CY-7 | 0.25 | eBioscience |
| CD3ε | 17A2 | Rat IgG2b, κ | APCe780 | 1 | eBioscience |
| CD44 | IM7 | Rat IgG2b, κ | eFluor450 | 0.5 | eBioscience |
| CD25 | PC61 | Rat IgG1, λ | Alexa700 | 1 | Biolegend |
| CD4 | GK1.5 | Rat IgG2b, κ | PE | 1 | eBioscience |
| CD8α | 53-6.7 | Rat IgG2a, κ | PE-Cy5 | 1 | eBioscience |
| FoxP3 | FJK-16s | Rat IgG2a, κ | PE-Cy5 | 1 | eBioscience |

APC = Allophycocyanin; IgG = Immunoglobulin; PE = R-Phycoerythrin
