# Supplementary material for: MMP-9 inhibition promotes anti-tumor immunity through disruption of biochemical and physical barriers to T-cell trafficking to tumors
Source: PLoS One. 2018 Nov 30;13(11):e0207255. doi: 10.1371/journal.pone.0207255 (PMC6267998; doi:10.1371/journal.pone.0207255)
Supplement: S3 Table — (DOCX) [file pone.0207255.s003.docx]

**Supplemental Table S3. Flow-cytometric gating strategy for each T-cell marker**

|  | **Gating downstream of singlet/live/CD45^+^ non-debris** |
| --- | --- |
| CD3^+^ T cells | CD3ε^+^ |
| CD8^+^ T cells | CD3ε^+^/CD8^+^CD4^-^ |
| CD4^+^ T cells | CD3ε^+^/CD8^-^CD4^+^ |
| Treg | CD3ε^+^/CD8^-^CD4^+^/CD25^+^FoxP3^+^ |
| CD8^+^CD44^+^ | CD3ε^+^/CD8^+^CD4^-^/CD8^+^CD44^+^ |
| CD4^+^CD44^+^ | CD3ε^+^/CD8^+^CD4^-^/CD4^+^CD44^+^ |

Treg = regulatory T cells
