# Supplementary figures and images for: MMP-9 inhibition promotes anti-tumor immunity through disruption of biochemical and physical barriers to T-cell trafficking to tumors
Source: PLoS One. 2018 Nov 30;13(11):e0207255. doi: 10.1371/journal.pone.0207255 (PMC6267998; doi:10.1371/journal.pone.0207255)

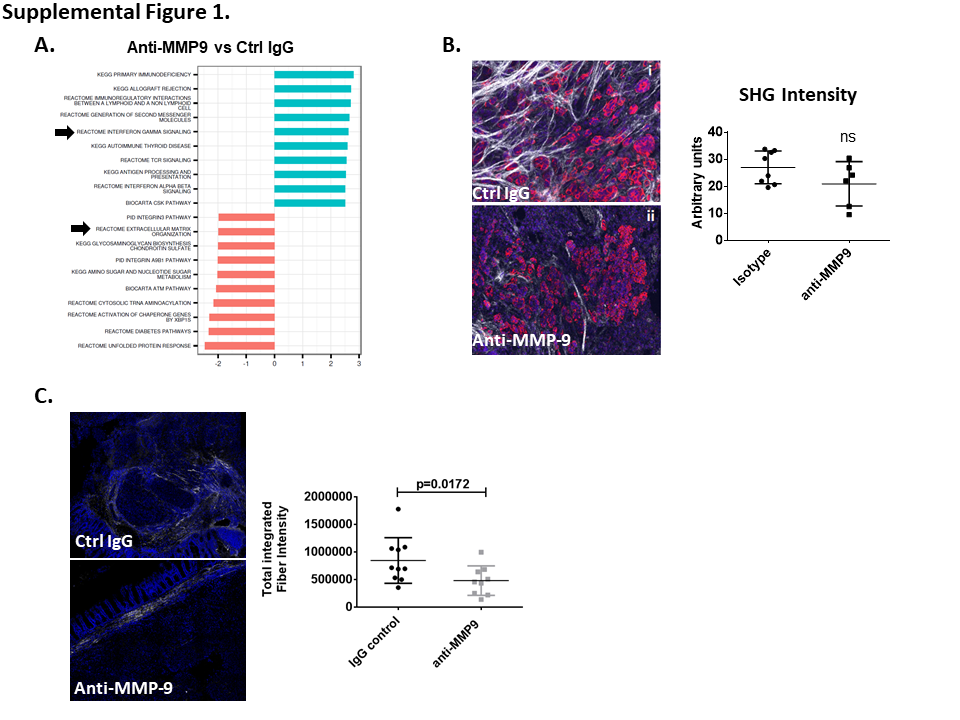

Supplement: S1 Fig — (A) Analysis of the top ten pathways modulated by anti-MMP9 revealed upregulation of pathways associated with immune activation and downregulation of ECM pathways (black arrows). (B) Consistent with RNAseq data, second harmonic imaging analysis of end-of-study tumors revealed that anti-MMP9–treated tumors showed a trend for decreased fibrillar collagen content as compared to control IgG–treated tumors. Figure shows results of analysis of the top 10 pathways altered by anti-PDL1 antibody treatment alone. (TIF) [file pone.0207255.s004.tif]

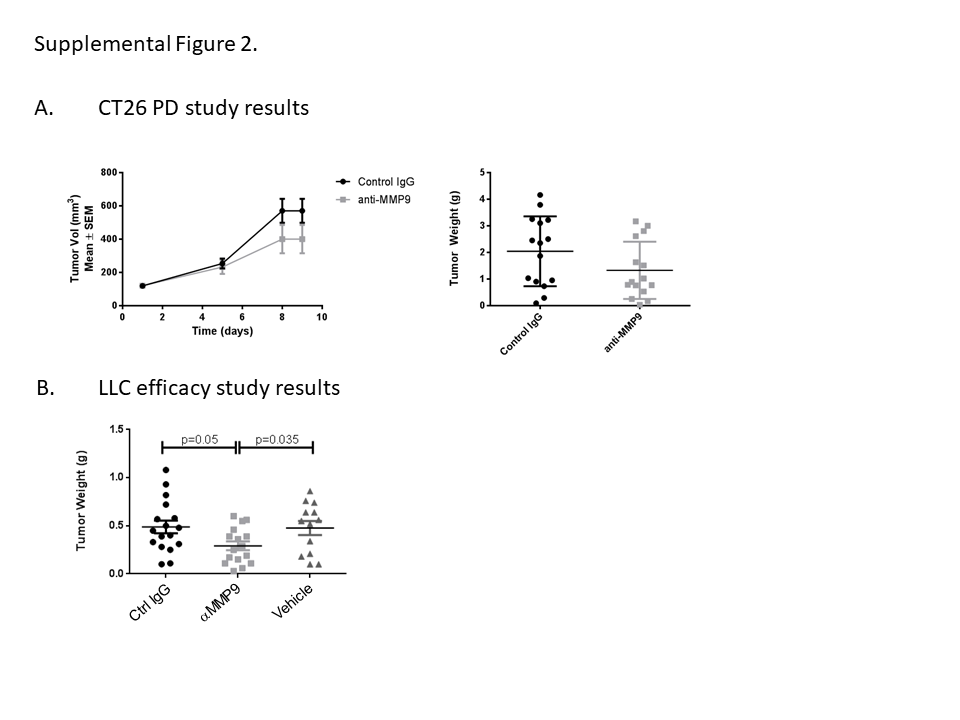

Supplement: S2 Fig — (A) Tumors from the CT26 orthotopic model were collected on day 10 for RNAseq analysis. Anti-MMP9 treatment showed no significant effects on tumor size at this time point. Tumor volume over the course of study (left panel); tumor weight at termination (right panel). (B) LLC tumors were collected at the end of study (day 14) for RNAseq. Mice treated with anti-MMP9 antibody showed a significant decrease in final tumor weight as compared to control IgG or vehicle treatment at time of gene expression analysis (p = 0.05). (TIF) [file pone.0207255.s005.tif]

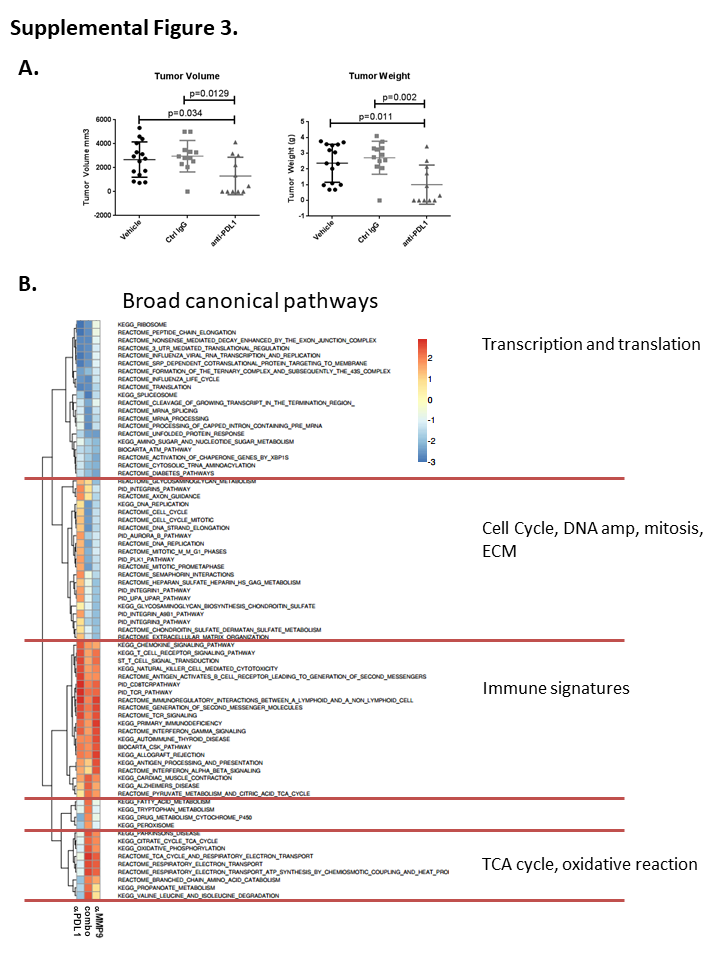

Supplement: S3 Fig — (A) Female BALB/c mice were injected orthotopically with RENCA tumor cells. Five days post-injection, animals were treated with 20 mg/kg anti-PDL1 twice weekly for 19 days. Treatment with anti-PDL1 antibody resulted in significant tumor growth reduction at the end of the study (p = 0.002). (B) RNAseq GSEA analysis of end-of-study NeuT tumor samples from study shown in Fig 3A. (TIF) [file pone.0207255.s006.tif]

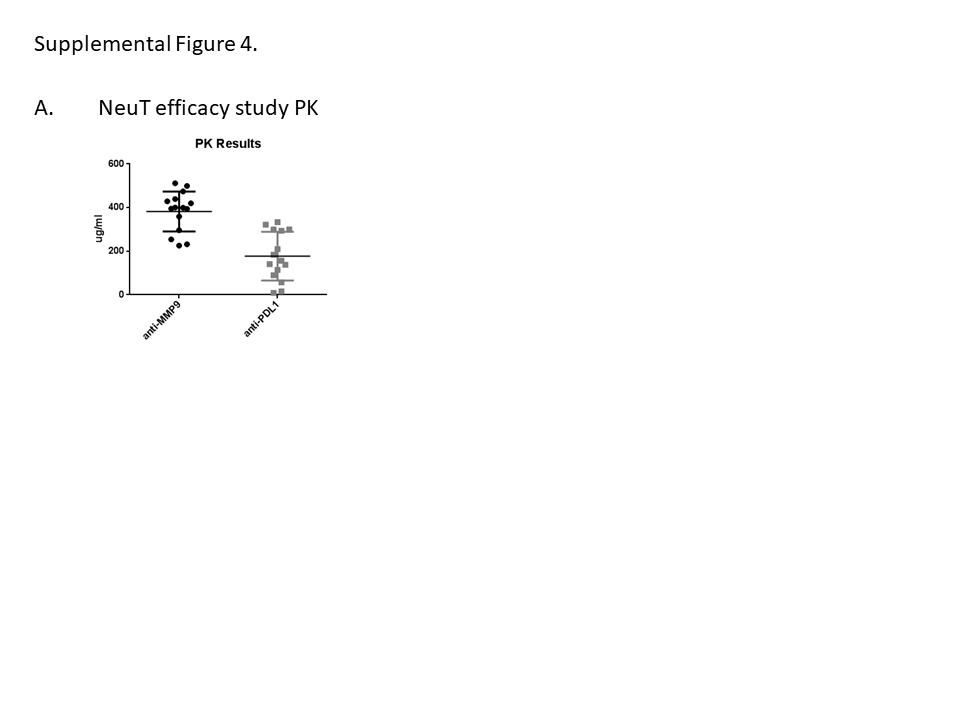

Supplement: S4 Fig — Serum was collected at the end of the study presented in Fig 3A. Both antibodies were found to be at sufficient levels to inhibit their respective antigen targets. (TIF) [file pone.0207255.s007.tif]
