# Supplementary material for: MMP-9 inhibition promotes anti-tumor immunity through disruption of biochemical and physical barriers to T-cell trafficking to tumors
Source: PLoS One. 2018 Nov 30;13(11):e0207255. doi: 10.1371/journal.pone.0207255 (PMC6267998; doi:10.1371/journal.pone.0207255)
Supplement: S1 File — (DOCX) [file pone.0207255.s008.docx]

# SUPPLEMENTAL MATERIALS

## Methods

For antibody reagents, in addition to HPLC-based purification, LAL (*Limulus* amoebocyte lysate) endotoxin testing (Endosafe; Charles River Laboratories, Charleston, SC) confirmed that the antibody preparation contained less than 5 EU/mg endotoxin.

For immunohistochemistry procedures: staining procedures were carried out using the Ventana Discovery Ultra Autostainer and associated reagents (Ventana Medical Systems, Tucson, AZ): Slides were deparaffinized with EZ Prep for 12 minutes at 69°C followed by CC1 cell conditioning for 64 minutes at 95°C. Slides were rinsed with Reaction Buffer, and 1 drop of ChromoMap Inhibitor was applied for 8 minutes, followed by Reaction Buffer rinse. Next, 100 uL of anti-MMP9 rabbit monoclonal antibody (ab76003; Abcam Inc, Cambridge, UK) at a concentration of 0.1 ug/mL was manually applied and incubated for 1 hour at room temperature, followed by Reaction Buffer rinse. A total of 1 drop of Anti-Rabbit HQ was then applied for 32 minutes, followed by Reaction Buffer rinse and 1 drop of Anti-HQ HRP for 32 minutes. Slides were then rinsed with Reaction Buffer and 1 drop of H_2_O_2_ conditioned medium (CM) was applied for 4 minutes, followed by 1 drop of DAB CM for 8 minutes. Slides were again rinsed with Reaction Buffer and 1 drop of Copper CM was applied for 4 minutes, followed by a Reaction Buffer rinse. Lastly, 1 drop of Hematoxylin II was applied for 12 minutes, followed by a final rinse in Reaction Buffer before being removed from the autostainer. Stained slides were dehydrated in a series of graded alcohol and cleared in 2 changes of xylene for 2 minutes each. Slides were then coverslipped using Dako Mounting Medium (Dako, Carpinteria, CA).

For flow cytometry procedures: Tumors were minced and incubated in a digestion buffer (1 mg/mL Collagenase A and 50 U/mL Deoxyribonuclease I in calcium and magnesium-free phosphate-buffered saline [PBS]) for 30 minutes at 37ºC. Digestion was quenched by FACS buffer (1 mM EDTA and 2% FBS in PBS), and a single-cell suspension was generated by passing through a cell 70 µm cell strainer. A total of 2 × 10^6^ cells per sample were used for immunostaining.
